# Delivery of spike-RBD by bacterial type three secretion system for SARS-CoV-2 vaccine development

**DOI:** 10.3389/fimmu.2023.1129705

**Published:** 2023-02-21

**Authors:** Yuchen Zhou, Jing Qu, Xiaomeng Sun, Zhuo Yue, Yingzi Liu, Keli Zhao, Fan Yang, Jie Feng, Xiaolei Pan, Yongxin Jin, Zhihui Cheng, Liang Yang, Un-Hwan Ha, Weihui Wu, Liang Li, Fang Bai

**Affiliations:** ^1^ State Key Laboratory of Medicinal Chemical Biology, Key Laboratory of Molecular Microbiology and Technology of the Ministry of Education, College of Life Sciences, Nankai University, Tianjin, China; ^2^ Department of Pathogen Biology, Shenzhen Center for Disease Control and Prevention, Shenzhen, China; ^3^ Intervention and Cell Therapy Center, Peking University Shenzhen Hospital, Shenzhen, China; ^4^ Peking University-The Hong Kong University of Science and Technology Medical Center, Shenzhen, China; ^5^ Department of Pharmacology, School of Medicine, Southern University of Science and Technology, Shenzhen, China; ^6^ Department of Biotechnology and Bioinformatics, Korea University, Sejong, Republic of Korea

**Keywords:** SARS-CoV-2 vaccine, *Pseudomonas aeruginosa*, live-attenuated, type 3 secretion system (T3SS), anti-virus immunity

## Abstract

COVID-19 pandemic continues to spread throughout the world with an urgent demand for a safe and protective vaccine to effectuate herd protection and control the spread of SARS-CoV-2. Here, we report the development of a bacterial vector COVID-19 vaccine (aPA-RBD) that carries the gene for the receptor-binding domain (RBD) of the SARS-CoV-2 spike protein. Live-attenuated strains of *Pseudomonas aeruginosa* (aPA) were constructed which express the recombinant RBD and effectively deliver RBD protein into various antigen presenting cells through bacterial type 3 secretion system (T3SS) *in vitro*. In mice, two-dose of intranasal aPA-RBD vaccinations elicited the development of RBD-specific serum IgG and IgM. Importantly, the sera from the immunized mice were able to neutralize host cell infections by SARS-CoV-2 pseudovirus as well as the authentic virus variants potently. T-cell responses of immunized mice were assessed by enzyme-linked immunospot (ELISPOT) and intracellular cytokine staining (ICS) assays. aPA-RBD vaccinations can elicit RBD-specific CD4^+^and CD8^+^T cell responses. T3SS-based RBD intracellular delivery heightens the efficiency of antigen presentation and enables the aPA-RBD vaccine to elicit CD8^+^T cell response. Thus, aPA vector has the potential as an inexpensive, readily manufactured, and respiratory tract vaccination route vaccine platform for other pathogens

## Introduction

To stop the ongoing COVID-19 pandemic caused by severe acute respiratory syndrome coronavirus 2 (SARS-CoV-2), several vaccines have been developed through various platforms, among which live-attenuated bacteria has been gaining attention as a versatile tool. The live-attenuated bacterial vaccine stands out for its fast and low cost, suitable for mass production, promising to be leveraged for a rapid emergency response. Moreover, the bacterial vectors of the vaccine, exemplified by Bacillus Calmette-Guérin (BCG), could promote non-specific cross-protection against other bacterial and viral infections (1–4). BCG is a live attenuated *Mycobacterium bovis* vaccine that is widely used to prevent tuberculosis (TB) and was among the most broadly used vaccinations in the 20th century in neonatal and young children (5, 6). It could lead to long-term activation and reprogramming of innate immune cells, engaging pattern recognition receptors (PRRs) and trained innate immunity (7–9). To date, several bacterial vaccines have been studied or in clinical trial phase: *Bifidobacterium longum* DNA vaccine (bacTRL-Spike) from Canada, *Salmonella typhimurium* expressing spike protein (S.T. Ag-e.spike) and *Mycobacterium paragordonae* expressing receptor binding domain (Mpg-RBD-7) candidate vaccines from Korea, *Francisella tularensis* (rLVS *ΔcapB*) candidate vaccine co-expressing spike, nucleocapsid and membrane proteins from U.S.A. (10–12). However, currently available vaccines possess shortcomings, such as inefficient protein delivery capacity to antigen presenting cells, and thus trigger a weak cell-mediated immune response, especially memory T cell response (13, 14). The intracellular delivery of proteins is challenging, our work nonetheless suggests that a T3SS-based delivery system of live-attenuated *Pseudomonas aeruginosa* (aPA) serving as a platform could translocate the desired proteins and elicit immune memory response.

T3SS is a naturally occurring protein transport nanomachinery, highly conserved among Gram-negative bacteria. Expression of the machinery and its effectors is triggered upon contact with the host cells or induced by low calcium environment, such as in the presence of calcium chelator EGTA *in vitro*. Effectors are translocated through T3SS injectisome which is a syringe-like nanomachine that could puncture the host cell membrane and inject the effectors directly into host cytosol, making it a promising tool for protein delivery directly into the target cells (15). Furthermore, the ease of bacterial genetic and physiological manipulations also made them extremely attractive for used in vaccine applications. When proteins of interests are fused with the secretion signal of a T3SS effector ExoS (S_54_) (16), and the strain *P. aeruginosa* was deleted of all its native T3SS effectors while maintaining a functional injectisome, the recombinant proteins can be efficiently injected into various cell lines such as A549, 5637, HL‐60, mESCs and hESCs (17). Notably, the *P. aeruginosa* also naturally colonizes in the lungs, conferring a convenient intranasal route to stimulation of tissue-resident immunity (18, 19). Hence, due to its excellent delivery ability and possibility of eliciting both CD4^+^ and CD8^+^ immune response, we developed a series of T3SS-based aPA vaccines, in which the T3SS effectors, secretion repressor, and several acute virulence factors were deleted, as well as a gene essential for growth to confer the auxotrophic phenotype.

Herein, to investigate the possibility of developing intranasal administered COVID-19 vaccines using the RBD of SARS-CoV-2 spike protein along with the already informed aPA strains, we constructed an expression plasmid, in which the RBD was fused behind the N-terminal secretion signal of the T3SS effector ExoS. The aPA strains harboring the plasmid were able to inject the fusion protein into host cells in a T3SS-dependent manner. Upon nasal delivery, the vaccine strain triggered potent cellular and antibody responses. The data provide a reference for preparing other attenuated bacterial vaccines.

## Materials and methods

### Bacterial strains and plasmids

The bacterial strains and plasmids used in this study are listed in **Table 1**, along with their description and sources. To ensure the stability of these genetically modified bacteria, a mandatory step in this study is that the bacteria need to be freshly streaked on selective plates from −80°C storage and the expression of the RBD was verified by western blotting before each experiment.

**Table 1:** *P. aeruginosa* strains and plasmids used in this study.

Strain and plasmid	Description	Source
*P. aeruginosa*		
PAK-J	wild type *P. aeruginosa* strain with enhanced T3SS	(16)
Δ*exsA*	PAK-J deleted of *exsA*, which encoded the master activator of *P. aeruginosa* T3SS	(20)
Δ*popD*	PAK-J deleted of T3SS translocon pore formation gene *popD*, which is essential for the protein injection	(16)
Attenuated *P. aeruginosa* (aPA)		
Δ3	PAK-J deleted of *exoS*, *exoT*, and *exoY*	(16)
Δ5	Δ3 deleted of *ndk*, and *popN*	This study
Δ6	Δ5 deleted of *murI* (D-Glu auxotroph)	This study
Δ8	Δ5 deleted of *lasR-I*, *rhlR-I* and *xcpQ*	(21)
Δ9	Δ8 deleted of *murI* (D-Glu auxotroph)	(22)
Plasmids		
pExoS_54_F	*Escherichia*-*Pseudomonas* shuttle expression plasmid, Cb^R^	(23)
pS_54_-RBD[wt]	pExoS54F fused with SARS-CoV-2 spike-RBD [YP_009724390.1 (R319-F541)] gene, Cb^R^	This study
pS54-RBD[Delta]	Similar to the RBD[wt] sequence, except for two mutations (L452R, T478K)	This study
pS_54_-RBD[BA.1]	Sequence containing 13 mutations (G339D, R346K, S371L, S373P, S375F, S477N, T478K, E484A, Q493R, G496S, Q498R, N501Y, Y505H), compared to that of RBD[wt]	This study

### Immunization of mice

Specific pathogen-free (SPF) 7-9 weeks old female C57BL/6 mice were purchased from Beijing Vital River Laboratory Animal Technology Co., Ltd. (licensed by Charles River). All mice used in this study are in good health and are not involved in other experimental procedure. Mice were housed with 5 companions per cage. All animals were allowed free access to water and standard chow diet and provided with a 12-hour light and dark cycle (temperature: 20-25°C).

To prepare inocula for infections, *P. aeruginosa* strains were grown in LB (Δ3, Δ5, Δ8) or LB with10 mM D-Glu (Δ6, Δ9) overnight, and then subcultured in fresh medium, grown at 37°C to an OD_600_ of 1.0. The cells were harvested by centrifugation and pellets washed twice were suspended in sterile 0.9% NaCl, adjusted to 5 × 10^8^ CFU/ml. For vaccination, mice were immunized with indicated aPA strains [with a total volume of 20 μl (5 × 10^7^ CFU bacteria) per mouse] *via* intranasal route at biweekly intervals. As an intranasal vaccination control, recombinant RBD protein (GenScript, Z03483) was diluted with PBS, and mixed with an equal volume of curdlan adjuvant (20 mg/mL) (24). Serum samples were collected after vaccination as indicated in figures legends.

### Tissue bacterial loads

To assess bacterial loads in lungs and spleens, mice immunized with Δ5 and Δ6 were euthanized at indicated time points for each experiment. Tissues were extracted aseptically, homogenized in sterile 0.9% NaCl and enumerated as colony forming units (CFU) by plating 10-fold serial dilutions on L-agar plates.

### Indirect ELISA

All wells in 96-well plates were coated with the recombinant RBD protein (1 μg/ml) in 0.05 M carbonate-bicarbonate buffer at 4°C overnight, and blocked by PBST (PBS containing 0.05% Tween 20) supplemented with 5% skim milk at 37°C for 3 h. Serum samples were 10-fold serial dilution and added to each well, followed by incubation at 37°C for 1 h. After being washed with PBST for five times, plates were incubated with anti-mouse IgG/HRP (Promega, USA) and detected with 3,3’,5,5’-tetramethylbenzidine (TMB) substrate (ACMEC, China). Reactions were stopped with 1 M sulphuric acid, and the absorbance was measured at 450 nm in an ELISA reader (Varioskan Flash, USA). The endpoint titer was defined as the highest reciprocal dilution of serum to give an absorbance greater than 2.5-fold of the background values.

### Pseudovirus neutralization assay

A lentivirus-based SARS-CoV-2 pseudovirus system [GenScript (Cat. No. SC2087A)] expressing a Spike protein on the surface (Accession number: YP_009724390.1) was generated according to the instruction manual. Briefly, neutralizing antibody activity is measured by assessing the inhibition of luciferase activity in HEK293 target cells expressing the ACE2 receptor, following preincubation of the pseudovirus with 5-fold serial dilutions of the serum specimen. Titers are reported as the highest reciprocal serum dilution at which the relative light units (RLUs) were reduced by greater than 50% compared with virus control wells.

### Live SARS-CoV-2 neutralization assay

Δ6-RBD vaccines induced neutralizing activities against live SARS-CoV-2 WT or variants infection were detected using the plaque assay as described previously (25). The experiment was conducted in a BSL-3 laboratory at Shenzhen Center for Disease Control and Prevention. In brief, serum from each immunized mouse was diluted and mixed with the same volume of SARS-CoV-2 (100 PFU) and incubated at 37°C for 1 h. Thereafter, 200 μL of the virus-serum mixtures were transferred to pre-plated Vero E6 cells in 24-well plates. Inoculated cells were incubated at 37°C for two days. Then, Vero E6 cells were fixed with 4% paraformaldehyde and permeabilized with 0.2% Triton X-100. The cells were incubated sequentially with primary antibody against the SARS-CoV-2 nucleocapsid (NP) (SinoBiological) overnight at 4°C, horse radish peroxidase (HRP)-conjugated secondary antibody (Abcam), and TMB substrate (KPL). The plaque reduction neutralizing antibody titer (PRNT_50_) was defined as the minimal serum dilution that suppressed > 50% of viral plaques.

### ELISPOT

To detect RBD-specific T lymphocyte response, an IFN-γ -based ELISPOT assay was performed. Mice spleens were collected and lymphocytes were isolated. 96-well plates were precoated with anti-mouse IFN-γ antibody overnight at 4°C and then blocked for 2 hours at room temperature. Different concentrations of the recombinant RBD protein were added to the well, and then lymphocytes were added to the plate (1.5 × 10^5^/well). Cell Activation Cocktail (without Brefeldin A) [BioLegend (Cat. No. 423301)] was added as a positive control and cells stimulated with 0.9% NaCl were employed as a negative control. After 24 hours of incubation, the cells were removed, and IFN-γ was captured by biotinylated detection antibody, streptavidin-HRP conjugate and AEC substrate.

### Flow cytometry

Approximately 1.5 × 10^6^ cells were stained with antibodies and antibody application was followed by the recommendation. Mouse lymphocytes were stimulated with the peptide pool of SARS-CoV-2 RBD and incubated with monensin [BioLegend (Cat. No. 420701)] for 9 hours. Then, the cells were harvested. For surface staining, cells were stained with fluorescence-labeled mAbs of CD3-FITC (BioLegend, USA), CD4-APC-Cy7 (BioLegend, USA) and CD8-AF700 (BioLegend, USA). The cells were subsequently fixed and permeabilized in permeabilizing buffer (BD Biosciences, USA) and intracellularly stained with fluorescence-labeled mAbs of IFN-γ-BV605 (BioLegend, USA), IL-2-BV421 (BioLegend, USA) and IL-4-PE (BioLegend, USA). All stained cells were detected on BD LSRFortessa™ X-20.

### Statistical analysis

Data are shown as mean ± SD. All calculations and statistical analyses were performed using GRAPHPAD PRISM 8.0.1 (GraphPad Software, USA) for Windows.

## Results

### Construction of aPA-RBD vaccine candidates

**Figure 1:**
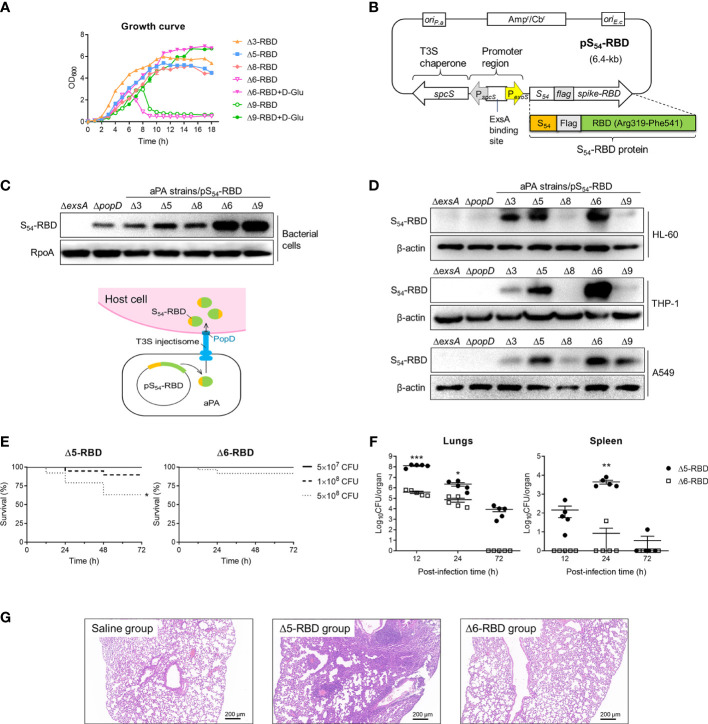
Construction and toxicity of candidate aPA-RBD vaccines against SARS-CoV-2. **(A)** Growth and viability of indicated attenuated *P. aeruginosa* (aPA) strains in LB liquid medium. D-Glu corresponds to 10 mM D-glutamate. **(B)** Expression vector of SARS-CoV-2 Spike-RBD fusing with the T3SS secretion signal S_54_ and a Flag tag on the N-terminal. ExsA is the master regulator for *P. aeruginosa* T3SS. **(C)** Identification of the fusion protein. Under 5 mM EGTA inducing conditions, aPA-RBD strains were examined for the ability to express the fusion protein by anti‐Flag immunoblot of the bacterial pellets. Antibacterial RpoA (bacterial RNA polymerase subunit) immunoblot was used as the bacterial internal reference. **(D)** Ability of bacterially injection. Human alveolar basal epithelial cell line A549, promyelocytic cell line HL-60, and monocytic cell line THP-1 were cocultured with indicated *P. aeruginosa* strains for 3 hours at MOI of 100, lysed and examined for protein injection by anti‐Flag immunoblot. β-actin was used as the internal reference of mammalian cells. **(E)** Mice survival after intranasal administration with different amounts of Δ5-RBD and Δ6-RBD strains. CFU, colony‐forming unit. Survival curves are generated by the Log-rank (Mantel-Cox) test to determine the statistical significance; *, *P*<0.05. **(F)** Bacterial loads in lungs and spleen from mice after vaccination with Δ5-RBD or Δ6-RBD strain (5 × 10^8^ CFU per mouse). Comparisons between Δ5-RBD and Δ6-RBD infected groups were performed by Student’s *t*-test (unpaired, two-tail); Error bars represent SD. *, *P* < 0.05; **, *P* < 0.01; ***, *P* < 0.001. **(G)** Hematoxylin and eosin **(HE)** staining of lung tissues collected at day 7 from immunized mouse. Scale bar = 200 μm.

We generated a series of attenuated *P. aeruginosa* strains (aPA) by successive gene deletions of the intrinsic T3SS effectors and repressor, as well as several acute virulence factors and an essential gene (**Table 1**). These deletion mutations were constructed on genomic loci that are not be flanked by active mobile elements or gene duplications (15, 26, 27).Due to the deletion of a glutamate racemase gene *murI*, aPA strains (Δ6 and Δ9) acquired an auxotrophic phenotype and had to grow in the presence of exogenous D-glutamate (D-Glu) (**Figure 1A**) (17, 28). The spike-RBD (R319 to F541) of SARS-CoV-2 was fused behind the N-terminal 54 amino acids of ExoS (S_54_) and expressed in *P. aeruginosa* on an expression plasmid pS_54_-RBD (**Figure 1B**). Various aPA strains harboring the pS_54_-RBD were subjected to T3SS induction by 5 mM EGTA for 3 h. As shown in **Figure 1C**, the S_54_-RBD fusion protein was expressed in all of the aPA strains, but not in the T3SS-defective mutant strain (Δ*exsA*), indicating that the expression of S_54_-RBD is dependent on T3SS activation. Moreover, similar to our previous observation (17), the D-Glu auxotrophic aPA strains produced more of the S_54_-fusion proteins under the D-Glu depleted condition (**Figure 1C**). To further assess the capacity of aPA to deliver RBD into the human cells related to pulmonary infection, alveolar basal epithelial cell line A549, promyelocytic cell line HL-60, and monocytic cell line THP-1 were co-incubated with aPA strains of Δ3-RBD, Δ5-RBD, Δ6-RBD, Δ8-RBD or Δ9-RBD, individually, at MOI of 100 for 3 h. After removal of the bacterial cells, the human cells were examined for intracellular S_54_-RBD proteins by western blotting. As the results shown in **Figure 1D**, the S_54_-RBD could be translocated into human cells by aPA Δ5 and Δ6 the most efficiently. However, no translocated S_54_-RBD was detected in the cells following co-culture with Δ*exsA* or the injection deficient mutant Δ*popD* strains (**Figure 1D**), although the fusion was produced by the Δ*popD* strain (**Figure 1C**). These results indicated that intracellular delivery of the S_54_-RBD protein by aPA occurred in a T3SS-dependent manner, and aPA Δ5 and Δ6 exhibited high capability of antigen delivery.

### Safety of aPA-RBD in mice

To evaluate the safety of aPA-RBD, we first measured mice survival after intranasal inoculation with strains Δ5-RBD and Δ6-RBD. Using this model of acute lung infection, the LD_100_ (the minimal lethal dose for 100% of mice) of wild-type (wt) PAK-J strain was 2 × 10^7^ CFU. In contrast, the observed LD_100_ for the Δ5-RBD and Δ6-RBD strains were > 1 × 10^9^ CFU (**Supplementary Figure S1**). The survival rate of the Δ6-RBD group is obviously higher than that of the Δ5-RBD group. A 100% survival was observed in both Δ5-RBD and Δ6-RBD groups when the administration dose was 5 × 10^7^ CFU (**Figure 1E**). Consistently, bacterial loads in lungs and spleens of Δ6-RBD were significantly lower than those inoculated with Δ5-RBD (**Figure 1F**). As no D-amino acids are available in mammals, the D-Glu auxotroph Δ6-RBD was eliminated within 72 hours after intranasal administration of 5 × 10^8^ CFU bacterial cells, shorter persistence time than that of Δ5-RBD (**Figure 1F**).

Then, we also assessed the pathological manifestations of lungs in mice one week after vaccination. As shown in **Figure 1G**, compared to the saline group, the lung of the Δ5-RBD group exhibited a more severely distorted structure, with a larger number of inflammatory cells infiltration in the pulmonary interstitium. However, no obvious tissue damage was observed in the Δ6-RBD group. Collectively, these results suggest that the strain Δ6-RBD, absolutely requiring D-glutamate for growth and featured a stable auxotrophic phenotype, confers much lower virulence than that of strain Δ5-RBD. 16S rRNA gene sequencing analysis of intestinal and pulmonic samples of mice suggest that the diversities of microbiota in Δ5-RBD and Δ6-RBD groups were not significantly different from those of the blank and Δ6-vehicle groups when ignoring the differences exhibited within the groups (**Supplementary Figure S2**).

### Generation of antibody-mediated immune responses

**Figure 2:**
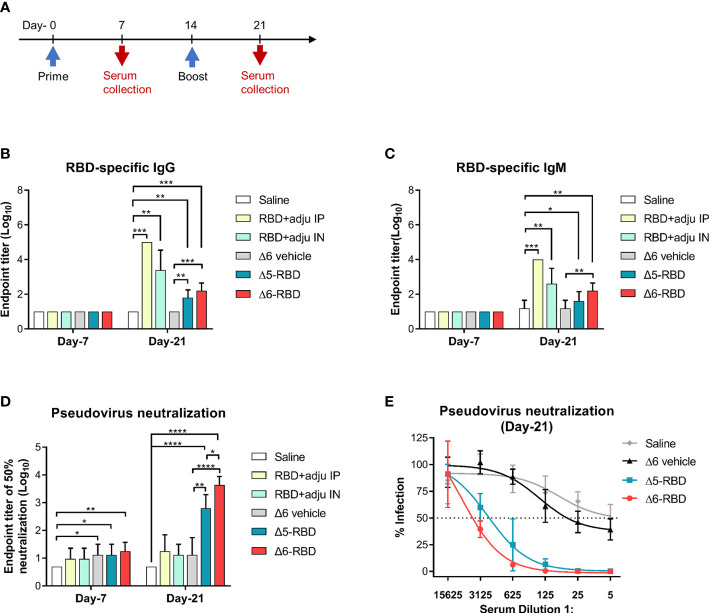
Characterization of aPA-RBD induced humoral immune response. **(A)** A prime-boost vaccination regimen was performed. Mice (n =5 per group) were immunized intranasally with 5 × 10 ^7^ CFU of the aPA-RBD vaccines (Δ5-RBD and Δ6-RBD) as well as the Δ6/pExoS_54_F (Δ6 vehicle) at days 0 and 14. 10-μg of recombinant RBD protein, with curdlan as adjuvant, was given intranasally (RBD+adju IN) or intraperitoneally (RBD+adju IP) as the RBD controls. Saline was given as sham control. Sera were collected 7 and 21 days after first immunization and assessed for specific antibody against SARS-CoV-2 Spike-RBD. **(B, C)** Anti-RBD IgG and IgM titers. Comparisons were performed by Student’s *t*-test (unpaired, two-tail); Error bars represent SD. *, *P* < 0.05; **, *P* < 0.01; ***, *P* < 0.001; ****, *P* < 0.0001. **(D)** Neutralization potency of aPA-RBD. Five-fold serial dilutions of immune serum from immunized mice was assessed for neutralizing and inhibiting the infectivity of SARS-CoV-2 pseudovirus. Pseudovirus neutralization assay shows the 50% neutralization titer (NT_50_). **(E)** Pseudovirus neutralization assay of Day-21 sera. The y-axis corresponds to observed percentage of pseudovirus infection in HEK293 cells that express human ACE2. The horizontal dashed line denotes 50% infection. The x-axis corresponds to reciprocal serum dilution.

To assess the immunogenicity of aPA-RBD, we immunized each C57BL/6 mice with the Δ5-RBD, Δ6-RBD, and empty Δ6/pExoS_54_F (Δ6 vehicle) by intranasal administration of 5 × 10^7^ CFU. 10-μg of recombinant RBD protein with the adjuvant curdlan in PBS was administered intranasally as a comparison, while saline was given as sham control. We performed a two-dose regimen to assess the response dynamics (**Figure 2A**). Mice sera were collected one week after each immunization and measured for the humoral responses. The presence of RBD-specific IgG and IgM antibodies was evaluated by indirect ELISA using SARS-CoV-2 Spike-RBD recombinant protein as coating antigen. Sera obtained 7 days after the second dose of the candidate vaccines showed elevated IgG and IgM against the recombinant RBD (**Figures 2B,C**). By contrast, the sera from control mice treated with saline or empty aPA (Δ6 vehicle) showed only background-level antibody responses. Notably, the recombinant RBD protein with curdlan (RBD control) was more effective in inducing the production of RBD-specific antibodies, especially the group (RBD+adju IP) immunized through the intraperitoneal route (**Figures 2B,C**).

Then, to investigate the capability of Δ5-RBD and Δ6-RBD on inhibiting the infectivity of SARS-CoV-2 pseudovirus, a neutralization assay was performed. Sera from both Δ5-RBD and Δ6-RBD groups resulted in a significant neutralization of the pseudovirus infectivity compared to those from the saline and Δ6 vehicle group. Furthermore, Δ6-RBD vaccination induced a higher neutralizing activity compared to the Δ5-RBD group on day 21 (**Figure 2D**), and the sera showed a strong 50% neutralization at a dilution > 1:3125 (**Figure 2E**). Interestingly, the groups immunized with intranasal and intraperitoneal inoculation of RBD protein plus adjuvant (RBD control) on day-21 showed low neutralizing activity (**Figure 2D**), although they elicit a high level of RBD-specific antibodies (**Figures 2B,C**), suggesting that Δ6-RBD group may have alternative ways enhancing the antibody-dependent neutralization (29).

### Neutralizing activity against Live SARS-CoV-2 in the sera of two-dose Δ6-RBD vaccinated mice

**Figure 3:**
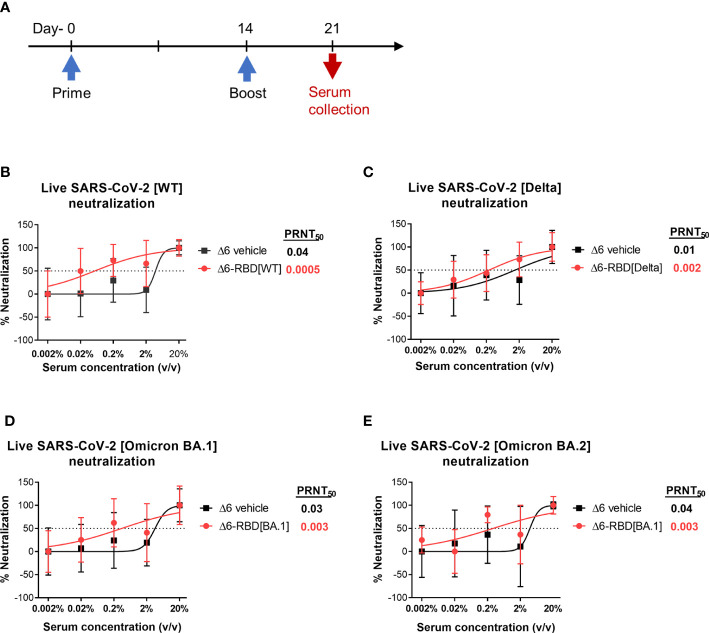
Assessment of aPA-RBD as vaccine against live SARS-CoV-2. **(A)** A prime-boost regimen was performed. Mice (n=6 per group) were vaccinated with Δ6-RBD[WT], Δ6-RBD[Delta] and Δ6-RBD[Omicron BA.1], and the sera were collected at 1 week after the second immunization. **(B, C)** Plaque reduction neutralization test (PRNT) assay of Δ6-RBD[WT] and Δ6-RBD[Delta] to wild‐type virus (Wuhan‐Hu‐1) and Delta variant. Fifty percent of the plaque reduction neutralizing antibody (PRNT_50_) titers against live SARS-CoV-2 were calculated as plaque reduction rate compared to Mpg (control) in Vero E6 cells. **(D, E)** PRNT50 of Δ6-RBD[Omicron BA.1] to Omicron BA.1 and Omicron BA.2. The dashed lines indicate the level of PRNT_50_.

To validate the neutralization results, we asked if aPA-RBD vaccinated sera also neutralize and inhibit the infectivity of authentic SARS-CoV-2 virus. A prime-boost regimen was performed as shown in **Figure 3A**. By the plaque reduction neutralization test (PRNT) assay, the Δ6-RBD group exhibited the average PRNT_50_ (defined as the highest serum dilution that resulted in >50% reduction in the number of virus plaques) of 0.0005, and the Δ6 vehicle group showed average PRNT_50_ of 0.04 (**Figure 3B**). The PRNT_50_ value of Δ6-RBD group was 80-fold lower than that of Δ6 vehicle group, indicating that Δ6-RBD vaccinations can significantly induce humoral immune response that neutralize and inhibit the infection of Vero cells by SARS-CoV-2 Wuhan‐Hu‐1 (wild‐type, WT). We further constructed the Δ6-RBD vaccines against SARS-CoV-2 delta and omicron BA.1 variants, respectively, and assessed the neutralizing activity against live SARS-CoV-2 delta, omicron BA.1 and BA.2 variants. The Δ6-RBD groups exhibited average PRNT_50_ of 0.002 to 0.003, and the Δ6 vehicle groups showed average PRNT_50_ of 0.01 to 0.04 (**Figures 3C-E**). Notably, the sera of the mice vaccinated with Δ6-RBD[BA.1] showed a cross-neutralizing activity against omicron BA.2 variant. Overall, these results demonstrate the neutralizing capacity of the Δ6-RBD vaccines against different SARS-CoV-2 variants.

### Activation of cell-mediated immunity

In patients, virus-specific CD4^+^ and CD8^+^ T cell responses are associated with milder disease, suggesting an involvement in protective immunity against COVID-19 (30, 31). Therefore, an ideal vaccine is expected to evoke both the humoral and cellular arms of the immune system (32).

**Figure 4:**
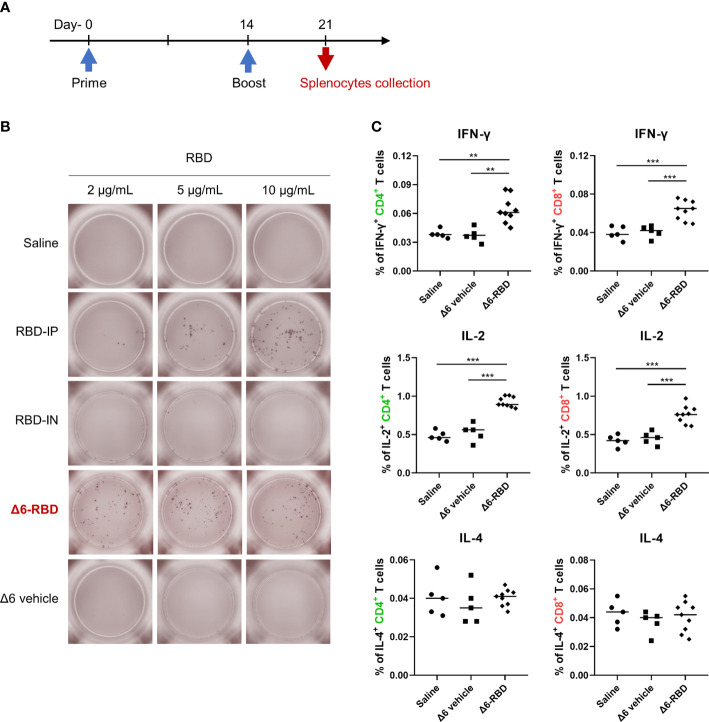
Induction of cellular responses by Δ6-RBD vaccination. **(A)** The vaccination regimen was performed. Mice were immunized at days 0 and 14, and spleens were isolated on day 21. The spleens soaked in 5 ml lymphocyte separation medium were dissociated by pressing it through the cell strainer (70 μm) using a syringe plunger. The splenocyte suspension was centrifuged at 800 x g for 30 min to obtain lymphocytes for cytokine detection. **(B)** ELISPOT analysis of lymphocytes. Cells were restimulated (20 hr) with recombinant RBD protein in the microwell of an ELISPOT plate that was precoated with the anti-mouse IFN-γ (5 μg/ml). Biotinylated anti-mouse IFN-γ (2 μg/ml) was used to detect the captured IFN-γ. Spots were visualized using Streptavidin-HRP enzyme and AEC substrate. **(C)** Cytokine profiling of lymphocytes by flow cytometry. Mice were immunized intranasally with 5 × 10^7^ CFU of the Δ6 vehicle (n=5) and Δ6-RBD (n=9), and lymphocytes restimulated (9 hr) with a peptide pool consisting of 20-mers (10 μg/ml) spanning the SARS-CoV-2-S RBD were detected for the expression of IFN-γ, IL-2 and IL-4 in CD4^+^ and CD8^+^ cells. *, *P*<0.05; **, *P*<0.01; ***, *P*<0.001.

To characterize the cellular immune responses, enzyme-linked immunospot (ELISPOT) and intracellular cytokine staining (ICS) assays were performed. Groups of Δ6-RBD vaccinated mice were sacrificed one week after second immunization (**Figure 4A**). To evaluate RBD-specific responses, lymphocytes derived from spleens were stimulated *in vitro* with different concentrations of the recombinant RBD protein (**Figure 4B**). Resultantly, cells from mice vaccinated with Δ6-RBD and the recombinant RBD protein (RBD-IP) yielded higher amounts of IFN-γ compared to the saline, RBD-IN, and Δ6 vehicle ones. The spot forming cells (SFC) of the RBD-IP group in response to the enhanced stimulation were increased, whereas that of the Δ6-RBD group appears to be concentration-independent.

We next studied RBD-specific CD4^+^ and CD8^+^ T cells by flow cytometric analysis after ICS. The lymphocytes of the Δ6-RBD group were stimulated with a pool of synthetic peptides covering the RBD domain of SARS-CoV-2 spike protein *in vitro* and the peptide-specific IFN-γ and IL-2 responses were observed in CD4^+^ and CD8^+^ T cells, while IL-4-secreting cells were not detectable in any of the immunization groups (**Figure 4C**), confirming that RBD-specific memory T cells are Th1-oriented. Similar to the ELISPOT results, the saline and Δ6 vehicle groups were unable to induce IFN-γ-producing T cells (**Figure 4C**). These results indicate that intranasal Δ6-RBD vaccination is able to activate both CD4^+^ and CD8^+^ T cell responses, and lead to RBD-specific Th1 skewed memory T cells responses.

## Discussion

The efficient delivery of SARS-CoV-2 spike RBD has been attributing to the bacterial T3SS of *P. aeruginosa*. The characteristic issue of proteins permeating cell membranes has been circumvented, and protein is directly delivered into target cells to stimulate an immune response, endowing the potential power of using bacterial T3SS-based antigendelivery as a vaccination method. However, the issues related to the detailed molecular mechanisms of proper folding and delivery of protein *via* the T3SS still need to be understood to broaden the application of this method. Here, both the auxotrophic strains Δ6 and Δ9 express high level of RBD (**Figure 1C**), whereas the Δ9 strain hardly delivers the RBD into HL-60 and THP-1 cells (**Figure 1D**). In contrast, it was reported that the Δ9 expresses a high level of Cre recombinase, and is capable of high‐efficiency protein delivery into HL-60 cell (17). This confirmed that the properties of the recombinant heterologous proteins affect the delivery efficiency, and it may exist the specific interplay between bacterial T3SS and host cells (33–35).

In addition to binding and directly interfering with viral entry, antibodies elicited by Δ5-RBD and Δ6-RBD may drive the neutralization of pseudovirus *via* their collaboration with the innate immune systems. As depicted in **Figures 2B,C**, adjuvants enhance the immune response to the two RBD-based subunit vaccines, especially of the group immunized *via* intraperitoneal injection. Intraperitoneal route could elicit a higher immunity and dissemination compared to the other ways such as oral gavage and aerosol (36, 37). Intriguingly, to the contrary of robust antibodies against RBD, groups vaccinated with RBD+adju IP and RBD+adju IN did not elicit a high neutralization titer against the SARS-CoV-2 spike protein to inhibit viral entry through the ACE2 receptor of HEK293 (**Figures 2D,E**), indicating that binding antibodies are not directly proportional to the neutralizing potency (38). Indeed, the accumulated interfering strength derives from the affinities of the binding antibody repertoire recognizing multiple epitopes of RBD, however, it displays a limited potency ceiling that can be surpassed by the assistance with innate immunity. One of the most striking illustrations is bacterial ghosts, an empty bacterial cell envelope retaining all the surface structural and antigenic components, which have an inherent immunogenicity and could function as both a vector and an adjuvant (39). Here, we postulate that the efficacy of Δ6-RBD may also be attributed to trained immunity. In particular, the complement system might enhance the neutralizing potency against SARS-CoV-2 (40, 41). To test this, we employed a pseudovirus assay for measuring antibody-mediated neutralization in the presence and absence of fresh serum (**Supplementary Figure S3**). The normal mouse serum (NMS) was heated at 56°C for 30 min to inactivate the complement system. We found that the neutralization potency of the heat-inactivated mouse serum (HIMS) was decreased compared to those of NMS. However, addition of fresh serum (HIMS + FS) had recovered, at least partially, on the neutralization by NMS. These results demonstrate that complement is capable of augmenting the neutralization potency of antibodies *in vitro*, which agrees with prior studies with respiratory syncytial virus (42, 43). Furthermore, recent study provides compelling evidence that the complement C4 could seal a virus through capsid inactivation, indicating the role of complement to arouse the vigor of neutralization.

In general, the main concern of using bacterial vector-based vaccines is the absence of glycosylation modification occurring in eukaryotes, such as the glycosylated spike protein of SARS-CoV-2 (44, 45). However, a previous study showed that the non-glycosylated RBD bacterial vaccine can induce a significant antibody with a neutralizing capacity even compared to a vaccination with glycosylated RBD with alum (12). It suggests that the immune response by non-glycosylated RBD bacterial vaccine could compensate for the reduced humoral immune response caused by the lack of glycosylation, which appears to be consistent with our results, promising that a considerable variety of antigens could be targeted by the bacteria-based vaccines.

To further explore the mucosal immunity, we measured the RBD-specific mucosal secretory IgA (S-IgA) in the bronchoalveolar lavage fluid (BALF) of the mice intranasal immunized twice with Δ5-RBD and Δ6-RBD on day 0 and day 14. No anti-RBD IgA was detected on day 7, day 21 or day 35. A possible explanation is that the T3SS mediated intracellular delivery of antigen tends to elicit RBD-specific IgG rather than IgA response. Further studies are needed to understand the mechanism, which might provide clues to enhance the protection efficacy.

It is known that RBD is the major target for NAbs interfering with viral receptor binding. In this context, we focused on the systemic immunity induced by the aPA-based vaccine. Extended studies should be performed to further evaluate the mucosal immunity elicited by the aPA-based vaccine, and compare the protection efficacy with commercialized COVID-19 vaccines.

During our study, we monitored the stability of the aPA bacteria. The vaccine strains were eliminated within three days following vaccination in mice (**Figure 1F**). After immunization, we inoculated the remained bacteria in culture medium with or without D-glutamate, and found that the bacterium remained as auxotrophic. These results demonstrate the stability and safety of the aPA strains. In the future, the vaccine efficacy needs to be examined in a proper animal model. The main impediment of the mouse model is the lack of appropriate receptors for effectively binding the spike protein and initiating viral infection. Herein, the vaccine-challenge studies in other animal models could be conducted subsequently, such as Syrian hamsters, ferrets, and non-human-primates. They are new options to develop quantifiable clinical symptoms, especially weight loss, hematological changes, and lung pathology, akin to humans seriously ill with COVID-19 (11, 46).

In conclusion, we generated an aPA-based SARS-CoV-2 vaccine candidate that elicits efficient T cell responses after primer-boost immunization, and high titers of NAb that may cross-react with new circulating variants. These promising data support the efficacy of the T3SS-based *P. aeruginosa* delivery system, highlight the feasibility for the development of the live auxotrophic vaccine platform.

## Data availability statement

The datasets presented in this study can be found in online repositories. The names of the repository/repositories and accession number(s) can be found in the article/**Supplementary Material**.

## Ethics statement

The animal study was reviewed and approved by the institutional animal care and use committee of the College of Life Sciences of Nankai University (permit number NK-04-2012).

## Author contributions

FB conceived the idea and ZY designed the experiments. YZ led the experiments and contributed to data analysis (with assistance from JQ). XS wrote the paper and all authors provided feedback. All authors contributed to the article and approved the submitted version.

## Supplementary material

Supplementary Figure 1 Mice survival after intranasal administration (1×10^9^ CFU) with Δ5-RBD and Δ6-RBD (n = 5).

Supplementary Figure 2 Beta diversities analysis of bacteria in feces (A) and lungs(B) among five groups [negative(N), PBS as the blank(B), empty Δ6(E), Δ5-RBD(5) and Δ6-RBD(6)] determined at day 21 after the second intranasal immunization (n = 3), estimated from 16S rRNA amplicon sequencing. The negative group of the mice was directly exported from specific-pathogen free (SPF) unit of laboratory animal company. Color reflects differences from low (blue) to high (red).

Supplementary Figure 3 Neutralization assay of serum collected on day 21. SARS-CoV-2 spike (WT) Fluc-GFP pseudovirus (Cat. No. PSSW-HLGB001) was cocultured with normal mouse serum (NMS), heat-inactivated mouse serum (HIMS) or HIMS replenished with fresh serum (HIMS + FS) for 60 min prior to infection of HEK293T cells that express human ACE2. Forty-eight hours later, cells were analyzed by PerkinElmer EnSpire for GFP expression. HIMS, heat inactivated at 56°C for 30 min.
