# Supplementary figures and images for: Delivery of spike-RBD by bacterial type three secretion system for SARS-CoV-2 vaccine development
Source: Front Immunol. 2023 Feb 21;14:1129705. doi: 10.3389/fimmu.2023.1129705 (PMC9988893; doi:10.3389/fimmu.2023.1129705)

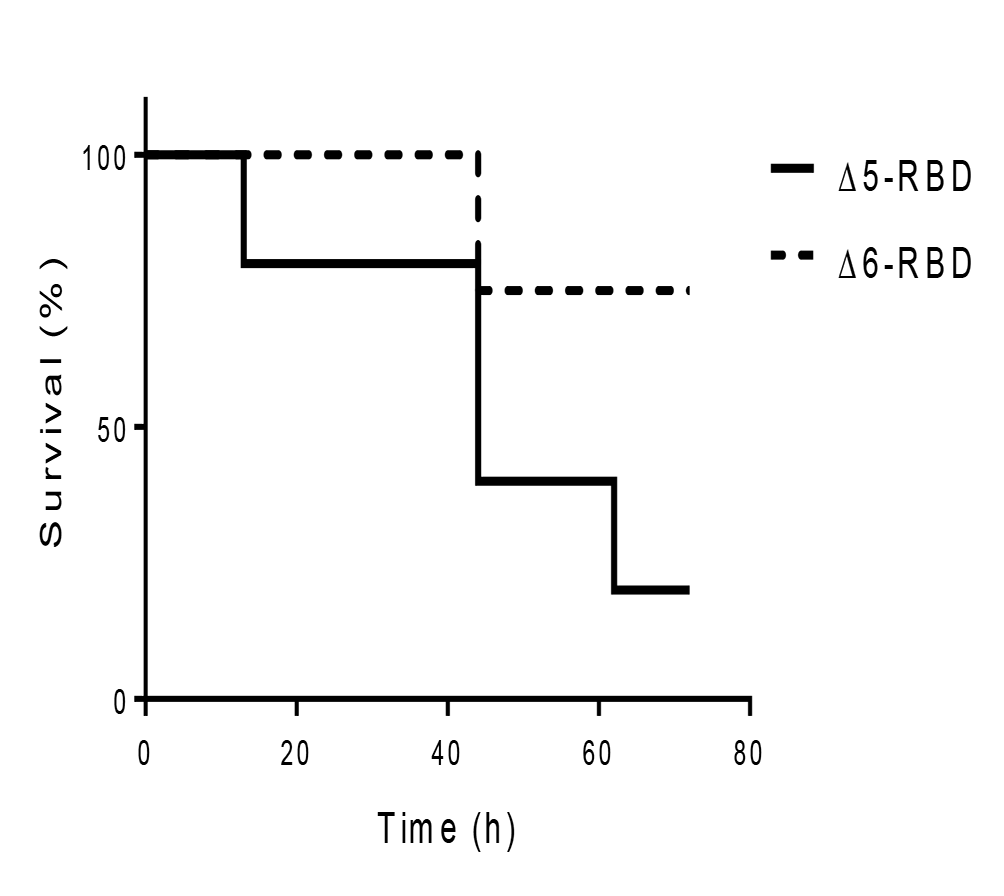

Supplement: Supplementary Figure 1 — Mice survival after intranasal administration (1×10^9^ CFU) with Δ5-RBD and Δ6-RBD (n = 5). [file Image_1.tif]

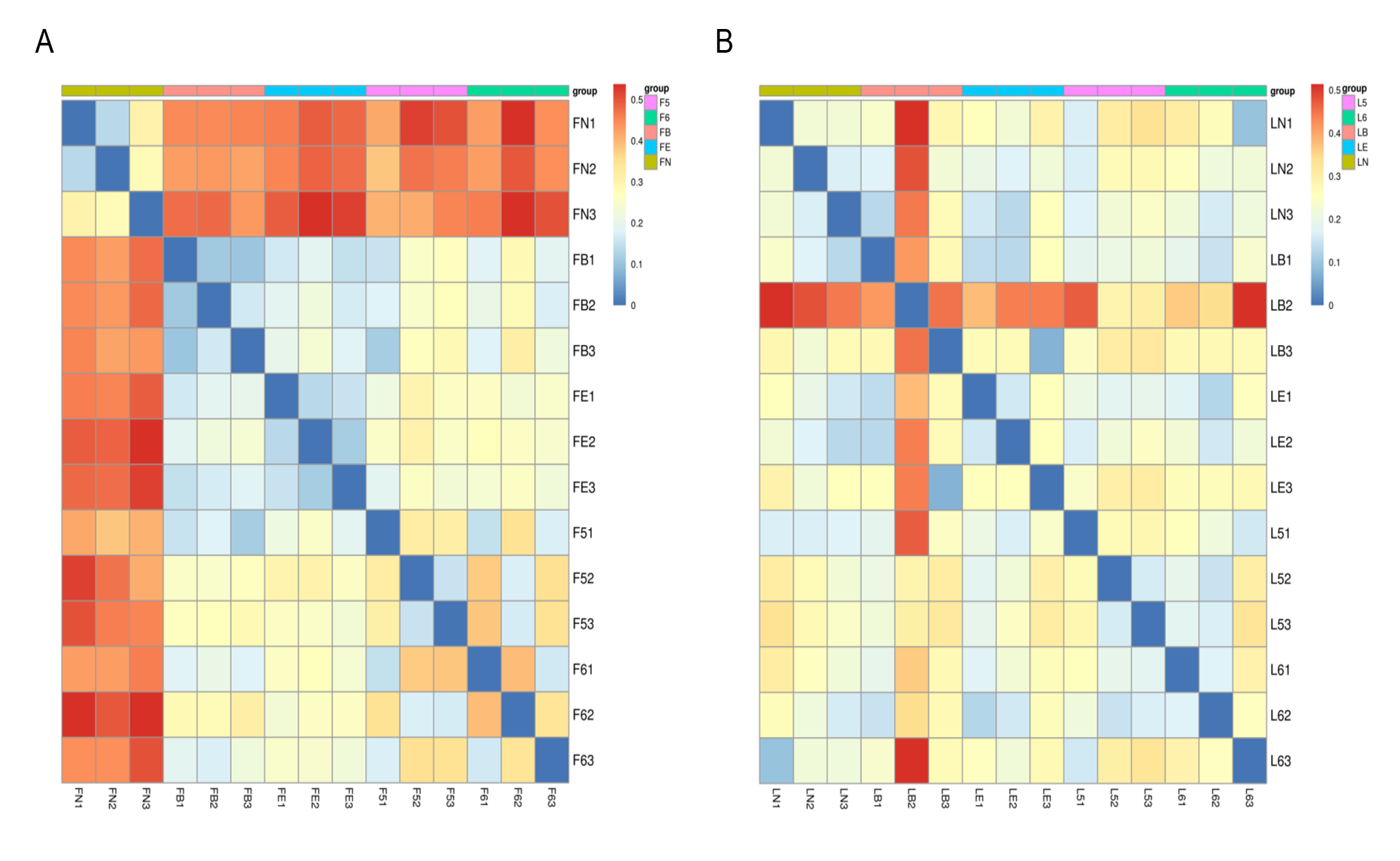

Supplement: Supplementary Figure 2 — Beta diversities analysis of bacteria in feces (A) and lungs(B) among five groups [negative(N), PBS as the blank(B), empty Δ6(E), Δ5-RBD(5) and Δ6-RBD(6)] determined at day 21 after the second intranasal immunization (n = 3), estimated from 16S rRNA amplicon sequencing. The negative group of the mice was directly exported from specific-pathogen free (SPF) unit of laboratory animal company. Color reflects differences from low (blue) to high (red). [file Image_2.tif]

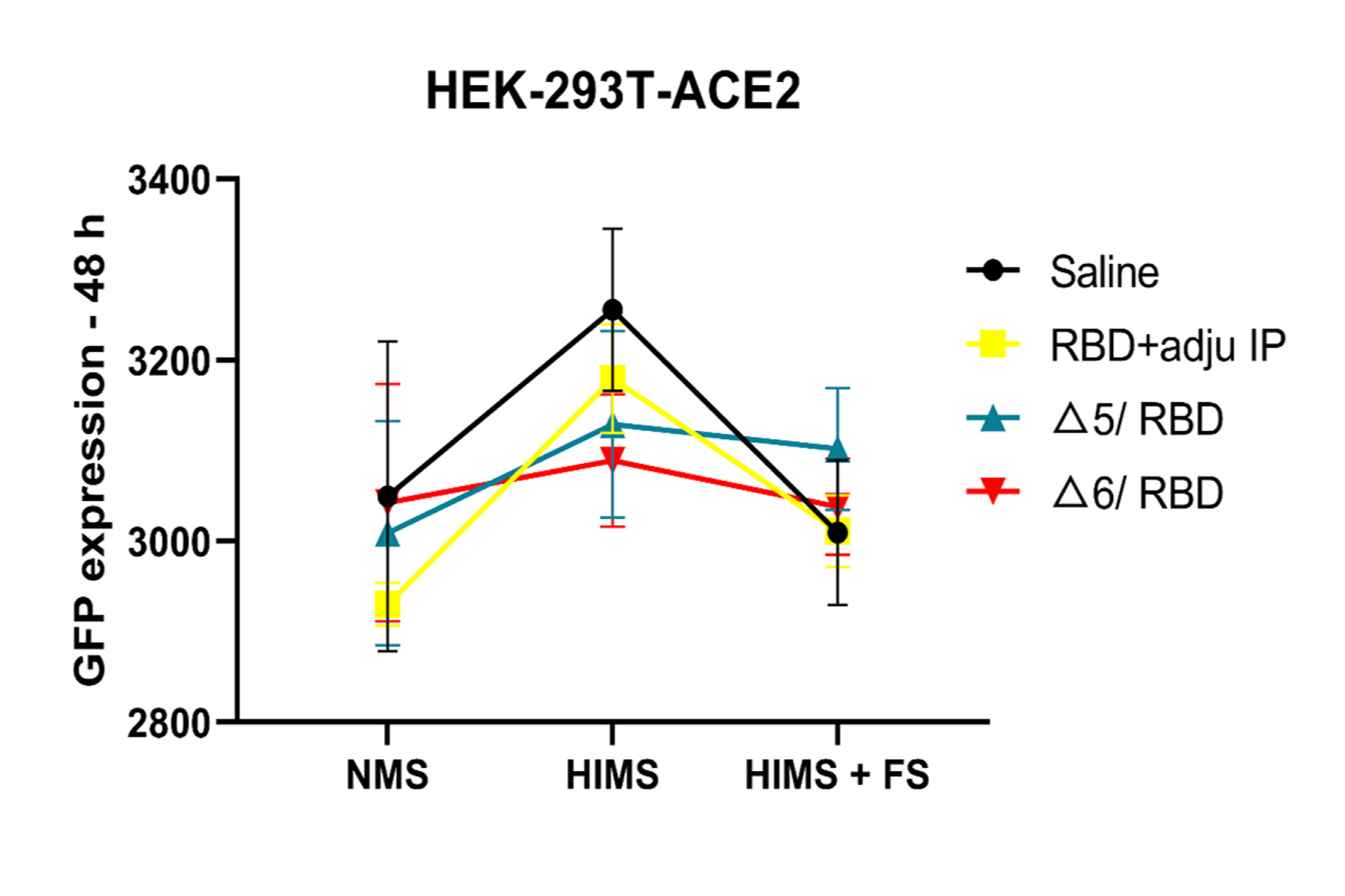

Supplement: Supplementary Figure 3 — Neutralization assay of serum collected on day 21. SARS-CoV-2 spike (WT) Fluc-GFP pseudovirus (Cat. No. PSSW-HLGB001) was cocultured with normal mouse serum (NMS), heat-inactivated mouse serum (HIMS) or HIMS replenished with fresh serum (HIMS + FS) for 60 min prior to infection of HEK293T cells that express human ACE2. Forty-eight hours later, cells were analyzed by PerkinElmer EnSpire for GFP expression. HIMS, heat inactivated at 56°C for 30 min. [file Image_3.tif]
